# Electrospun PAN/PVA-CS Membranes with Asymmetric Wettability for Simultaneous Emulsion Separation and Dye Removal

**DOI:** 10.3390/membranes16070224

**Published:** 2026-06-29

**Authors:** Tengfei Liao, Zengpeng Zhang, Qingxia Zhang, Hao Yang

**Affiliations:** Key Laboratory for Green Chemical Process, Ministry of Education, School of Environmental Ecology and Biological Engineering, Wuhan Institute of Technology, Wuhan 430205, China

**Keywords:** electrospinning, asymmetric wettability, superoleophobic, chitosan, emulsion separation, dye removal

## Abstract

Multifunctional membranes capable of simultaneously separating oil–water emulsions and removing organic dyes from complex aqueous systems have garnered considerable attention in recent years. However, the facile fabrication of high-performance membranes that integrate both separation and adsorption functions remains a significant challenge. Herein, we report the fabrication of a polyacrylonitrile/polyvinyl alcohol–chitosan (PAN/PVA-CS) bilayer membrane with asymmetric wettability via electrospinning. The micro/nanostructures and surface wettability of the as-prepared membranes were precisely tailored by modulating the chitosan (CS) concentration. The resultant PAN/PVA-CS membrane exhibited an overall separation efficiency exceeding 97.5% for mechanically emulsified samples. Notably, the PVA-CS layer demonstrated superhydrophilicity and excellent underwater oleophobicity, enabling the gravity-driven simultaneous separation of oil-in-water emulsions and adsorption of water-soluble Congo red dye without requiring external pressure. The maximum adsorption capacity for Congo red reached 61.3 mg g^−1^, surpassing that of numerous reported membrane-based and adsorbent materials. Concurrently, the hydrophobic PAN layer in the bilayer structure enabled the separation of water-in-oil emulsions. Overall, this work provides a promising strategy for the rational design of asymmetrically wettable multifunctional membranes with great potential for practical application in the purification of complex industrial wastewater containing both emulsified oils and soluble organic dyes.

## 1. Introduction

The uncontrolled discharge of oily wastewater from industrial and food-processing operations is a serious threat to global aquatic ecosystems, such as rivers, lakes, and oceans [1,2]. This wastewater often contains highly stable oil–water emulsions [3,4] and water-soluble contaminants such as organic dyes, which collectively endanger human health and ecological security [5,6,7,8]. Consequently, the development of multifunctional membranes capable of simultaneously separating oil–water emulsions and removing organic dyes has attracted significant research interest for advanced wastewater treatment [9,10].

To achieve this dual functionality, membranes must be rationally designed with tailored surface wettability and an abundance of functional groups (e.g., amino and carboxyl groups) for effective dye–molecule interaction. For instance, Zhang et al. [11] Fabricated a PVDF/chitosan/dopamine composite membrane via a 24 h immersion process; the membrane demonstrated. Notably, the PVA-CS layer demonstrated superhydrophilicity and excellent underwater oleophobicity, which enabled the gravity-driven simultaneous separation of oil-in-water emulsions and adsorption of water-soluble Congo red dye without requiring external pressure, anti-oil adhesion, and self-cleaning properties, enabling the separation of oil-in-water emulsions containing organic dyes (5 mg L^−1^). Similarly, a nanostarch-modified filter paper prepared by a facile spraying method exhibited switchable superhydrophilicity. Notably, the PVA-CS layer demonstrated superhydrophilicity and excellent underwater oleophobicity, which enabled the gravity-driven simultaneous separation of oil-in-water emulsions and adsorption of water-soluble Congo red dye without requiring external pressure and could remove methyl blue (10 mg L^−1^) during emulsion separation [12]. Bao et al. [13] also reported a Co_3_O_4_/TiO_2_/GO-coated copper mesh, fabricated by vacuum deposition, which displayed superamphiphilicity in air and superamphiphobicity under both water and oil, facilitating the separation of emulsions containing Congo red (20 mg L^−1^). Despite these advances, most reported membranes involve complex fabrication processes that hinder large-scale production. More critically, their dye adsorption capacities remain relatively low, severely limiting their practical utility for treating wastewater with high dye concentrations.

Electrospinning has emerged as a promising technique for fabricating high-performance oil–water separation membranes owing to its operational simplicity, precise control over membrane wettability and structure, and excellent scalability [14,15]. The interconnected three-dimensional fibrous network of electrospun membranes provides abundant sites for demulsification, which is crucial for separating highly stable emulsions [16,17,18]. However, electrospun membranes specifically designed for the simultaneous separation of oil-in-water (O/W) emulsions and the adsorption of organic dyes are still scarce [19]. A key challenge is the rational design of electrospun fibrous membranes with synergistically optimized wettability, hierarchical structure, and surface chemistry to achieve efficient and concurrent emulsion separation and dye adsorption.

Chitosan (CS) is a natural polysaccharide that has attracted considerable attention for environmental remediation, especially for oil–water separation, because of its biodegradability, biocompatibility and non-toxicity [20]. It has rich amino and hydroxyl groups and is promising for use as an adsorbent for various organic dyes [21]. However, fabricating pure CS nanofibers via electrospinning is extremely challenging due to their polycationic nature in aqueous solutions, which results in high viscosity and poor spinnability [22]. Furthermore, pure CS membranes often suffer from limited chemical and mechanical stability under harsh conditions [23].

A common method to address these disadvantages is to mix CS with other spinnable polymers, such as polyvinyl alcohol (PVA) [24], polyethylene oxide (PEO) [25], and polylactic acid (PLA). PVA is especially attractive because of its good hydrophilicity, biodegradability, and compatibility with CS [26,27]. The blend of PVA and CS not only enhances spinnability and stability but also introduces hierarchical roughness, which is beneficial for producing superwetting surfaces. However, freestanding CS-PVA nanofiber mats usually do not have sufficient mechanical strength for long-term use [28]. The construction of a bilayer membrane is effective, in which a strong electrospun substrate provides mechanical support, and a functional CS-PVA nanofiber layer achieves the required surface properties.

Polyacrylonitrile (PAN) is a good base material because of its excellent electrospinnability, high mechanical strength, and chemical stability. In particular, CS can form strong hydrogen bonds with PAN, which can maintain a firm interface and help in the formation of a tough, hierarchical membrane [29]. To address the drawbacks of conventional separation membranes, such as poor spinnability and weak mechanical properties of pure chitosan nanofibers, complicated fabrication procedures, limited scalability and poor adaptability to complex wastewater, this work constructs an asymmetric wettable PAN/PVA-CS bilayer membrane via sequential electrospinning. The as-fabricated membrane achieves integrated bidirectional separation of oil-in-water and water-in-oil emulsions as well as simultaneous dye removal, breaking the limitation of a single separation function for traditional membranes. Hydrogen bonding interactions between chitosan, PVA and PAN polymer chains greatly strengthen interlayer bonding, fundamentally overcoming the poor formability and insufficient mechanical strength of pure chitosan electrospun fibers. The facile and controllable fabrication route shows great potential for large-scale industrial production. By precisely tuning the chitosan content, hierarchical micro-nano rough structures are purposely constructed, endowing the membrane with outstanding dye adsorption capacity while maintaining ultrahigh oil–water separation flux. Moreover, the bilayer membrane possesses excellent resistance to acid, alkali and salt corrosion, which can be applied to treat industrial wastewater with complex components and greatly broadens the practical application scope of multifunctional water purification membranes. This study offers a novel technical strategy for the facile and scalable fabrication of high-performance integrated composite water purification membranes.

## 2. Materials and Methods

### 2.1. Materials

Polyvinyl alcohol 1788 (PVA) and Congo red (CR) were purchased from Aladdin Reagent Co., Ltd. (Shanghai, China). Chitosan (CS, medium viscosity) was obtained from Macklin Biochemical Co., Ltd. (Shanghai, China). Polyacrylonitrile (PAN, Mw = 90,000) was supplied by Zhangmutou Huachuang Plastic Raw Materials Co., Ltd. (Dongguan, China). Glacial acetic acid, acetone, hexane, cyclohexane, petroleum ether, N, N-dimethylformamide (DMF), sodium chloride, sodium hydroxide (NaOH), and 30 wt% hydrogen peroxide (H_2_O_2_) were provided by Sinopharm Chemical Reagent Co., Ltd. (Shanghai, China). A 25 wt% aqueous glutaraldehyde solution and silicone oil were purchased from Fuyu Fine Chemical Co. Ltd. (Tianjin, China). Concentrated sulfuric acid (98 wt%) and hydrochloric acid (HCl, 35 wt%) were obtained from Comio Chemical Reagent Co., Ltd. (Tianjin, China). Edible vegetable oil was acquired from a local supermarket. All chemicals were of analytical grade and used without further purification unless otherwise stated.

### 2.2. Fabrication of PAN/PVA-CS Bilayer Membranes via Electrospinning

Preparation of PAN Substrate: A pristine PAN nanofiber membrane was first fabricated as the substrate. Briefly, 12 g of PAN powder was dissolved in 88 mL of DMF under magnetic stirring for 12 h at room temperature to obtain a homogeneous 12 wt% PAN solution. Electrospinning was performed using a syringe pump (LSP02-2B, Longer Pump, Baoding, Hebei) at a feeding rate of 1 mL h^−1^, an applied voltage of 20 kV, a tip-to-collector distance of 20 cm, and a rotation speed of 150 rpm. The process was conducted under ambient conditions (25 ± 2 °C, 40–50% relative humidity).

Fabrication of the PVA-CS Functional Layer: To prepare the functional layer, 4 g of PVA and predetermined amounts of CS were dissolved in 100 mL of a 20 vol% aqueous acetic acid solution under vigorous stirring at 95 °C for 6 h. After cooling to room temperature, the homogeneous PVA-CS solution was electrospun directly onto the prepared PAN substrate. The electrospinning parameters for the PVA-CS layer were as follows: applied voltage, 25 kV; feeding rate, 1 mL h^−1^; tip-to-collector distance, 15 cm; drum rotation speed, 150 rpm; and deposition time, 5 h. The relative humidity was maintained at 30–40% during this process.

The as-fabricated bilayer membranes were cross-linked by immersion in a 5 wt% aqueous glutaraldehyde solution for 30 min at room temperature to enhance their stability in aqueous environments. Subsequently, the membranes were thoroughly rinsed with deionized water (3 × 500 mL) to remove residual cross-linker and dried overnight in a forced-air oven at 50 °C. The resulting membranes were denoted as PPC5, PPC10, PPC20, and PPC30, corresponding to PVA/CS mass ratios of 100:5, 100:10, 100:20, and 100:30, respectively.

### 2.3. Oil–Water Mixture Separation Performance

The separation performance was evaluated using a laboratory-built, gravity-driven setup. Membrane samples (2.5 cm × 2.5 cm) were pre-wetted with the corresponding continuous phase and fixed between two polytetrafluoroethylene (PTFE) flanges, providing an effective separation area of 3.14 × 10^−4^ m^2^ (diameter: 2 cm).

For the oil–water mixture separation, 20 g of oil (hexane, cyclohexane, petroleum ether, or vegetable oil) and 10 g of deionized water were mixed and poured into the upper glass tube. The separation was solely driven by gravity. The time required for complete separation and the mass of the collected permeate were recorded. The separation efficiency (*η*, %) and permeation flux (*J*, L m^−2^ h^−1^) were calculated using Equations (1) and (2), respectively:

*η* = (*m*_1_*/m*_0_) × 100% (1)

*J* = *V*/(*A* × Δ*t*) (2)

where *m*_0_ and *m*_1_ are the masses of oil in the original mixture and collected filtrate, respectively. where *V* (L) is the volume of the permeate, *A* (m^2^) is the effective membrane area, and Δ*t* (h) is the permeation time.

### 2.4. Chemical Stability Tests

Chemical stability was assessed by evaluating the separation performance in corrosive media and the long-term stability after immersion. For the separation performance in corrosive media, oil–water mixtures were prepared using hexane or vegetable oil as the dispersed phase and 1 M HCl, 1 M NaOH, or 1 M NaCl solution as the continuous phase. The separation efficiency and flux were then measured as described in Section 2.3. For the long-term stability test, the membrane samples were immersed in 1 M HCl, 1 M NaOH, or 1 M NaCl solutions for 24 h at room temperature. After immersion, the samples were rinsed with deionized water until the filtrate reached a neutral pH, and their separation efficiency for a hexane/water mixture was re-evaluated.

### 2.5. Emulsion Separation Experiments

#### 2.5.1. Emulsion Preparation

Oil-in-water (O/W) emulsions were prepared by adding 1 mL of oil (hexane, cyclohexane, vegetable oil, petroleum ether, or silicone oil) to 99 mL of deionized water, followed by sonication at 300 W for 1 h. Water-in-oil (W/O) emulsions were prepared by adding 1 mL of deionized water to 99 mL of vegetable oil and sonicated under identical conditions.

#### 2.5.2. O/W Emulsion Separation

The PAN/PVA-CS membrane was pre-wetted with deionized water and mounted with the PVA-CS layer facing upward. A 50 mL aliquot of the O/W emulsion was poured onto the membrane, and separation proceeded under gravity. The oil concentrations in the feed (*C*_0_) and filtrate (*C*_1_) were determined using a UV–vis spectrophotometer (PerkinElmer Lambda-35, Waltham, MA, USA) [30,31]. Separation efficiency was calculated using Equation (3):

(3) $$\eta =(1-\frac{C_{1}}{C_{0}})\times 100\%$$

where *C*_0_ and *C*_1_ are the oil concentrations in the original emulsion and filtrate, respectively.

#### 2.5.3. W/O Emulsion Separation

For the W/O emulsion separation, the membrane was pre-wetted with vegetable oil and mounted with the PAN layer facing upward. The separation procedure and efficiency calculation were identical to those used for the O/W emulsions.

In all the emulsion separation tests, the height of the emulsion column was maintained at 15 cm. The permeation flux was calculated using Equation (2) by recording the time required for 10 mL of the filtrate to permeate.

### 2.6. Dye Adsorption Experiments

Congo red (CR) was used as the model anionic dye. All batch adsorption experiments were conducted in a constant-temperature shaker (ZhiCheng ZHWY-211B, Shanghai, China) at 25 °C and 200 rpm. Typically, 0.05 g of the membrane was immersed in 10 mL of a CR solution at a predetermined concentration. At specified time intervals, the residual dye concentration was measured at *λ_max_* = 499 nm using UV–vis spectrophotometry. (PerkinElmer Lambda-35, Waltham, MA, USA).

The equilibrium adsorption capacity (*Q_e_*, mg g^−1^) and dye removal efficiency (*R*, %) were calculated using Equations (4) and (5), respectively.

(4) $$Q_{e}=\frac{C_{0}-C_{e}}{m}\times V$$

(5) $$R=(1-\frac{C_{e}}{C_{0}})\times 100\%$$

where *C*_0_ and *C_e_* (mg L^−1^) are the initial and equilibrium CR concentrations, respectively, *m* (g) is the mass of the membrane sample, and *V* (L) is the volume of the dye solution.

For the adsorption kinetics, the initial CR concentration was fixed at 50 mg L^−1^. Solution samples were collected at prescribed time intervals (10–150 min) to determine the residual concentration, and the adsorption capacity at time *t* (*Q_t_*) was calculated using Equation (4).

### 2.7. Separation of Dye-Containing O/W Emulsions

A CR-containing hexane-in-water emulsion was prepared by mixing 99 mL of a 50 mg L^−1^ CR aqueous solution with 1 mL of hexane, followed by sonication at 300 W for 1 h. The optimal PPC20 membrane was pre-wetted with water and mounted with the PVA-CS layer facing upward for gravity-driven separation.

### 2.8. Characterization

The membrane morphology was characterized using field-emission scanning electron microscopy (FE-SEM, Zeiss GeminiSEM 300, Oberkochen, Germany). The samples were sputter-coated with platinum prior to imaging. The chemical composition was analyzed using Fourier transform infrared spectroscopy (FTIR, Thermo Fisher Nicolet 6700, Waltham, MA, USA) with an ATR accessory (4000–600 cm^−1^). The crystalline structure was examined by X-ray diffraction (XRD, Bruker D8 Discover, Billerica, MA, USA) with Cu Kα radiation (*λ* = 1.5406 Å, 30 kV, 10 mA, 2θ = 3–90°). Static water contact angles (WCA) and underwater oil contact angles (UOCA) were measured using an automatic contact angle goniometer (DataPhysics OCA 20, Filderstadt, Germany). For UOCA, a membrane sample immersed in water was probed with a 5 µL oil droplet. The reported values are the averages of at least three measurements at different locations. The emulsion and filtrate were characterized using digital photography and optical microscopy (Olympus CX43, Tokyo, Japan). The concentration of the dye in the aqueous solution was measured using a UV–vis spectrophotometer (PerkinElmer Lambda-35, Waltham, MA, USA). The concentration was determined using the peak height at 499 nm and a calibration curve (Figure S4a, R^2^ = 0.999).

## 3. Results and Discussion

### 3.1. Chemical Characterization and Morphological Analysis

**Figure 1:**
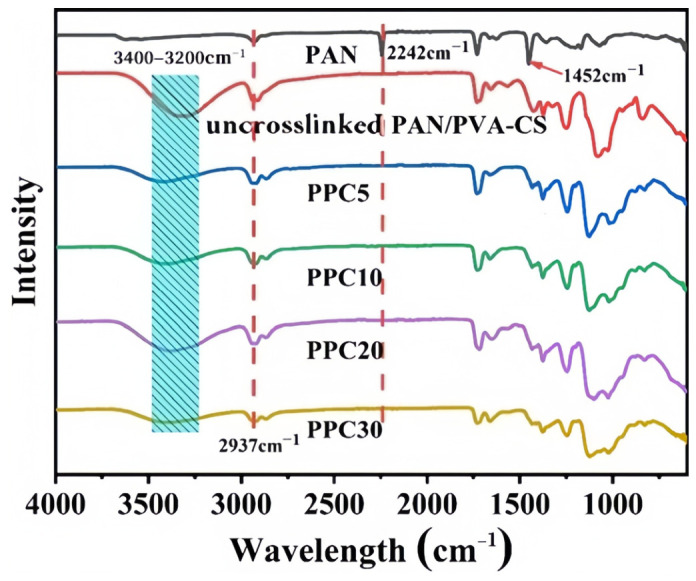
ATR-FTIR spectra of the pristine PAN membrane, uncrosslinked PAN/PVA-CS (PPC20) membrane, and crosslinked PAN/PVA-CS bilayer membranes with varying PVA-to-CS mass ratios (denoted as PPC5, PPC10, PPC20, and PPC30).

ATR-FTIR spectra were acquired for the pristine PAN membrane, uncrosslinked PAN/PVA-CS membranes, and crosslinked PAN/PVA-CS membranes with varying PVA/CS mass ratios. As shown in Figure 1, the spectrum of the pristine PAN membrane exhibited three characteristic absorption peaks at 2937, 2242, and 1452 cm^−1^, corresponding to aliphatic C–H stretching, C≡N stretching, and C–C skeletal vibrations, respectively [32]. For all PAN/PVA-CS bilayer composite membranes, a broad absorption band appeared in the range of 3200–3400 cm^−1^. This band originated from the overlap of the O–H and N–H stretching vibrations of PVA and CS, respectively. Notably, the O–H stretching band of PVA shifted to lower wavenumbers (near 3200 cm^−1^) compared to that of pure PVA. This shift provides direct spectroscopic evidence for the formation of extensive intermolecular hydrogen bonds between CS and PVA [33]. Additionally, two weak peaks at 2922 cm^−1^ and 2854 cm^−1^, attributable to the aliphatic C–H stretching vibrations of CS, were observed in the spectra of PAN/PVA-CS membranes.

After cross-linking with glutaraldehyde (GA), a new characteristic peak emerged at approximately 1660 cm^−1^. This peak was assigned to the C=N stretching vibration of the Schiff base formed via the nucleophilic addition reaction between the primary amino groups of CS and the aldehyde groups of GA. In contrast, the uncrosslinked PAN/PVA-CS membrane displayed only a distinct peak at 1567 cm^−1^, which is characteristic of the amide II band (N–H bending vibration) of CS. Overall, these FTIR results confirm the successful deposition of the PVA-CS functional layer onto the PAN substrate and the effective cross-linking reaction between CS and GA.

**Figure 2:**
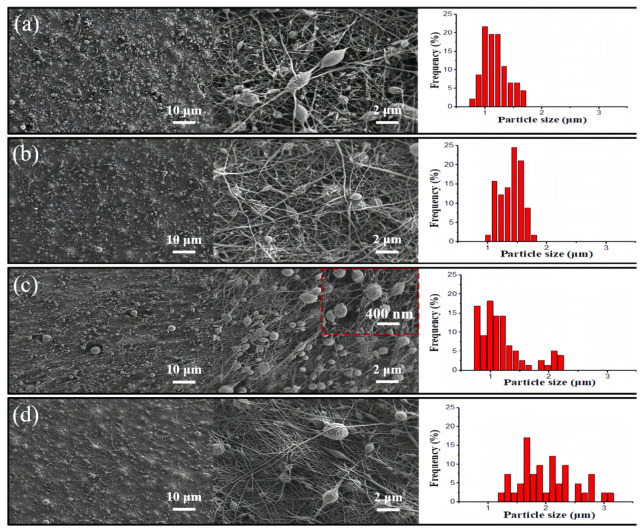
FE-SEM images and corresponding particle size distributions of PAN/PVA-CS bilayer membranes prepared with different PVA-to-CS mass ratios: (**a**) PPC5, (**b**) PPC10, (**c**) PPC20, and (**d**) PPC30.

The surface morphologies of the PAN/PVA-CS bilayer membranes with varying CS contents were characterized using field emission scanning electron microscopy (FE-SEM), as shown in Figure 2. The PPC5 membrane (Figure 2a), which had the lowest CS content, exhibited a surface dominated by large spindle-shaped structures with an average diameter of approximately 1 μm, accompanied by a relatively sparse fibrous network. When the CS content was increased to 10% (PPC10, Figure 2b), the density of spherical particles on the fiber surfaces increased significantly, and the overall fiber density was also enhanced [34]. The optimal microstructure was achieved at a CS content of 20% (PPC20; Figure 2c). The surface of the PPC20 membrane exhibited a distinctive multiscale architecture consisting of continuous nanofibers and uniformly dispersed spherical/spindle-shaped particles with sizes ranging from 0.5 to 2 μm. Particularly, the spindle-shaped features were not smooth but were assembled from smaller nanoparticles, thereby forming a secondary rough texture. In contrast to PPC5 and PPC10, PPC20 displayed a broader particle size distribution, collectively creating a hierarchical micro/nanostructure analogous to the lotus leaf surface. A further increase in the CS content to 30% (PPC30, Figure 2d) resulted in a more distinct phase separation between PVA and CS. Large spindle-shaped structures with diameters of 1–3 μm became the predominant surface characteristic, and radial nanofibrils were observable on the surfaces of these larger particles. In general, the average particle diameter on the surface increased monotonically with the CS content, and the size distribution of PPC20 and PPC30 increased. These observations suggest that by tuning the CS content to a sufficiently low level, electrospun membranes with a suitable hierarchical micro/nanostructure can be constructed in this bioinspired, lotus leaf structure. The hierarchical fibrous-particle structure provides abundant sites for demulsification, where oil droplets can be captured, gathered, and separated from water [35].

The cross-sectional morphology of the membranes was examined by SEM (Figure S1). As shown in Figure S1a, the membrane exhibited an asymmetric bilayer structure. The top PVA-CS functional layer had a uniform thickness of 6.15 μm (Figure S1b). At higher magnification (Figure S1c), the surface was observed to consist of spindle-like structures interconnected with fibers, while the underlying PAN substrate displayed smooth fibers and provided mechanical support.

### 3.2. Surface Wettability Analysis

**Figure 3:**
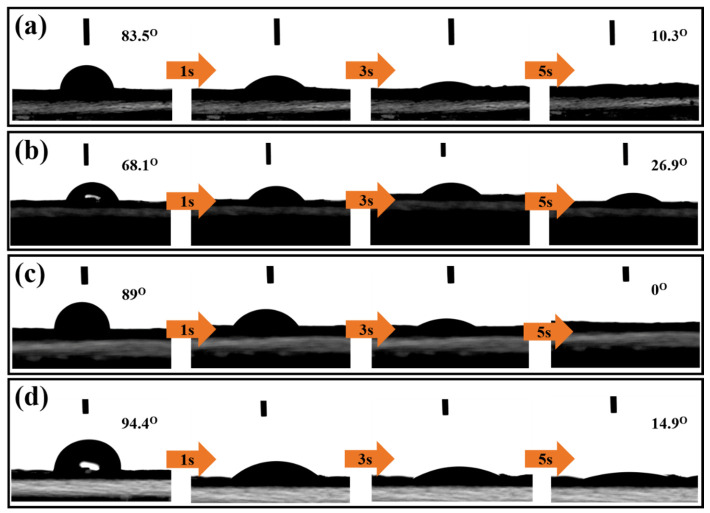
Time evolution of water contact angles on the PVA-CS surfaces of PAN/PVA-CS bilayer membranes with varying PVA-to-CS mass ratios: (**a**) PPC5, (**b**) PPC10, (**c**) PPC20, and (**d**) PPC30.

Surface wettability, typically assessed through contact angle measurements, is a critical parameter governing the separation performance of oil–water separation membranes [36]. Figure 3a–d presents the time-dependent evolution of the water contact angle (WCA) on the PVA-CS surfaces of the PAN/PVA-CS membranes with different CS contents. As shown, all PVA-CS surfaces exhibited rapid water spreading; however, the time required for the WCA to decrease to 0° varied significantly among the samples. The PPC20 membrane achieved complete water spreading within only 5 s, the shortest time observed, indicating its superior surface hydrophilicity. This excellent hydrophilic performance is primarily attributed to the well-defined hierarchical micro/nanostructure of PPC20, as confirmed by the FE-SEM results shown in Figure 2. The uniformly distributed multiscale particles on the PPC20 surface established a regular rough architecture that enhanced the capillary wicking effect. This effect promotes rapid water penetration into the membrane and facilitates the formation of a continuous hydration layer via hydrogen bonding, thereby leading to superhydrophilicity [34]. Additionally, the abundant hydroxyl (-OH) and amino (-NH_2_) groups on the PVA and CS chains provide numerous sites for hydrogen bonding with water molecules [37,38]. It should be noted that the intramolecular and intermolecular hydrogen bonds between PVA and CS may initially reduce the number of accessible functional groups. However, upon contact with water, these internal hydrogen bonds can be disrupted, allowing the functional groups to reorient toward the aqueous phase, further enhancing surface hydrophilicity.

The macroscopic appearance and wettability of the as-prepared membranes are shown in Figure S2. As shown in Figure S2c, the PAN/PVA-CS bilayer membrane exhibited a uniform, smooth, and large-area surface, indicating the good scalability of the sequential electrospinning process. This simple fabrication method is beneficial for reducing the time and cost of large-scale membrane fabrication. As shown in Figure S2a, the methylene blue-stained water droplets and oil red O-stained oil droplets placed on the PVA-CS surface of PPC20 both spread quickly, confirming the amphiphilic nature of the surface in air. In addition, the oil droplets on the surface of the PPC20 membrane were almost spherical when the membrane was immersed in water (Figure S2b), indicating the remarkable. Notably, the PVA-CS layer demonstrated superhydrophilicity and excellent underwater oleophobicity, which enabled the gravity-driven simultaneous separation of oil-in-water emulsions and adsorption of water-soluble Congo red dye without requiring external pressure on the PVA-CS layer. The sequential electrospinning method enables the fabrication of large-area membranes with uniform surface morphology. As shown in Figure S2, a free-standing PAN/PVA-CS bilayer membrane measuring approximately 50 cm × 30 cm was successfully prepared, demonstrating the potential of this approach for scale-up production. The ability to produce large, defect-free membranes on a laboratory scale suggests promising translatability to industrial manufacturing.

To quantitatively assess the underwater anti-oil adhesion properties of the optimal PPC20 membrane, a dynamic oil adhesion test was performed. The process of injecting a 3 μL oil red O-stained hexane droplet onto the fully water-prewetted PVA-CS surface of PPC20 was recorded using a high-speed camera. As shown in Figure S3, the oil droplet completely rebounded from the membrane surface upon contact, and no residual oil adhesion was observed. This result confirms that the PPC20 membrane possesses excellent underwater anti-fouling performance against oils, which is crucial for preventing membrane pore blockage and maintaining stable separation efficiency during long-term oil–water separation.

**Figure 4:**
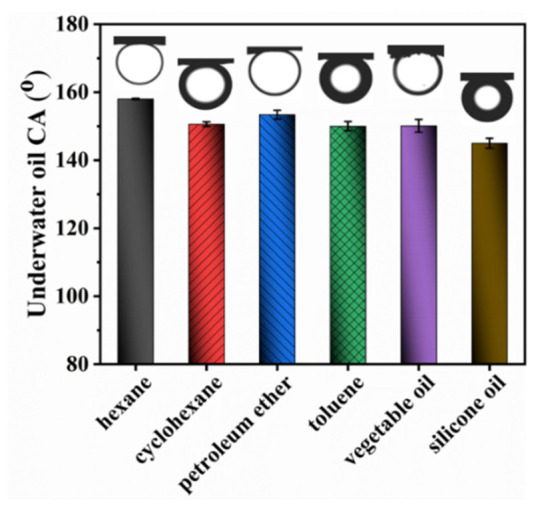
Underwater oil contact angles (UOCAs) of various oils on the PVA-CS functional layer of the optimal PPC20 membrane.

The underwater oil contact angles (UOCAs) of six typical oils and organic solvents on the PVA-CS surface of PPC20 are summarized in Figure 4. All the tested liquids exhibited strong underwater oil repellency, with UOCAs exceeding 145°. Hexane showed the highest UOCA (158° ± 1°), followed by petroleum ether (153° ± 1°), cyclohexane (151° ± 1°), vegetable oil (150° ± 1°), and toluene (148° ± 1°). Silicone oil presented the lowest UOCA of 145°± 1°. Notably, even for highly viscous silicone oil, the membrane maintained effective underwater oil repellency and was able to repel oil droplets without adhesion.

### 3.3. Study on the Separation Performance of Oil–Water Mixtures

**Figure 5:**
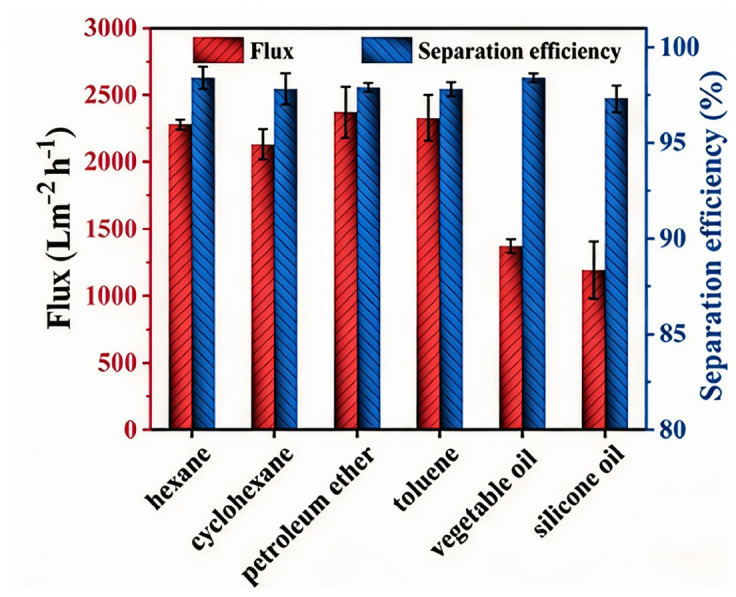
Separation efficiencies and permeation fluxes of various oil–water mixtures for the optimal PPC20 membrane, measured on the PVA-CS functional layer under gravity-driven conditions.

The gravity-driven separation performance of the optimal PPC20 membrane was evaluated for various light oil–water mixtures using the PVA-CS layer as the separating surface (Figure 5). The membrane demonstrated excellent separation efficiency, exceeding 97% for all the tested mixtures. The highest permeation flux, 2372.5 ± 189.6 L m^−2^ h^−1^, was achieved for the petroleum ether/water mixture, followed by the silicone oil/water mixture (2329.3 ± 170.8 L m^−2^ h^−1^). The vegetable oil/water mixture yielded the lowest flux of 1192.2 ± 213.1 L m^−2^ h^−1^. The reduced flux for high-viscosity oils (vegetable oil and silicone oil) was attributed to the formation of a thicker oil layer on the membrane surface, which increased the hydraulic resistance to water permeation.

**Figure 6:**
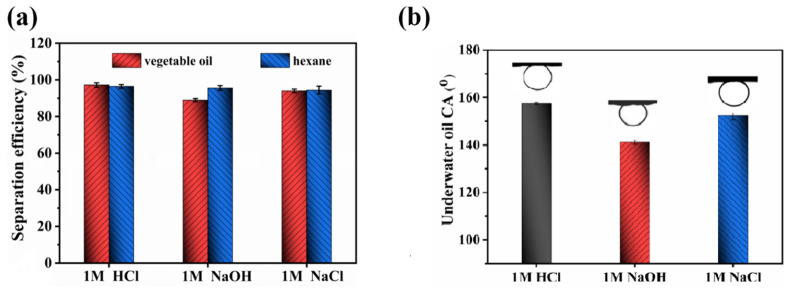
(**a**) Separation efficiencies of the optimal PPC20 membrane for various oil–water mixtures in acidic (1 M HCl), alkaline (1 M NaOH), and saline (1 M NaCl) media. (**b**) Underwater oil contact angles (UOCAs) of the PPC20 membrane after 24 h of immersion in acidic, alkaline, and saline solutions.

The chemical stability of separation membranes is crucial for practical applications, as oily wastewater often contains strong acids, alkalis, and high-salinity solutions [39]. Therefore, the chemical stability of the PPC20 membrane was assessed by examining its separation performance in corrosive aqueous environments and after long-term immersion in water. As shown in Figure 6a, the membrane maintained separation efficiencies above 96% for oil–water mixtures containing 1 M HCl, 1 M NaOH, or 1 M NaCl. Specifically, the separation efficiency for the vegetable oil/water mixture in acidic medium was the highest (≈97.1%), whereas that for the hexane/water mixture under the same conditions reached ≈96.4%. The efficiency in the alkaline medium was slightly lower than that in the acidic and saline media. This minor decline can be attributed to the slight swelling of the PVA-CS layer in a strong alkaline solution, leading to a modest increase in the inter-fiber spacing [40].

The underwater oil contact angles (UOCAs) of PPC20 after 24 h of immersion in acidic, alkaline, and saline solutions are presented in Figure 6b. The corresponding UOCAs were 158° ± 1°, 141° ± 1°, and 153° ± 2°, respectively. Notably, the UOCA decreased from 158° ± 1° to 141° ± 1° after alkaline treatment, which is consistent with the observed slight reduction in the separation efficiency.

### 3.4. O/W Emulsion Separation Performance

**Figure 7:**
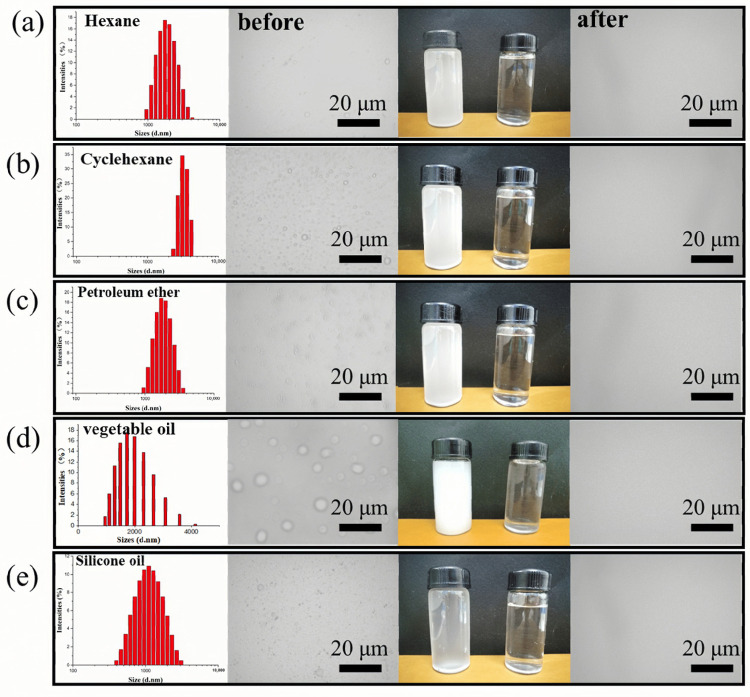
Digital images, corresponding optical micrographs, and droplet size distributions of various oil-in-water (O/W) emulsions before and after gravity-driven separation using the optimal PPC20 membrane: (**a**) hexane-in-water, (**b**) cyclohexane-in-water, (**c**) petroleum ether-in-water, (**d**) vegetable oil-in-water, and (**e**) silicone oil-in-water.

Oil–water emulsions are considerably more challenging to separate than immiscible oil–water mixtures due to their microscopic droplet size and high thermodynamic stability [41]. Therefore, the effective separation of stabilized emulsions significantly extends the practical application potential of membrane materials. The separation performance of the PPC20 membrane for different O/W emulsions was studied in this study. The as-prepared hexane-in-water and petroleum ether-in-water emulsions (Figure 7a,c) contained many oil droplets in the range of 1000–2000 nm. The initial milky-white emulsions became clear and transparent after gravity filtration, indicating effective emulsion separation. This was further confirmed by optical microscopy, which revealed no detectable oil droplets in the corresponding filtrates (Figure 7a,c). Comparable separation results were obtained for the other O/W emulsions tested.

**Figure 8:**
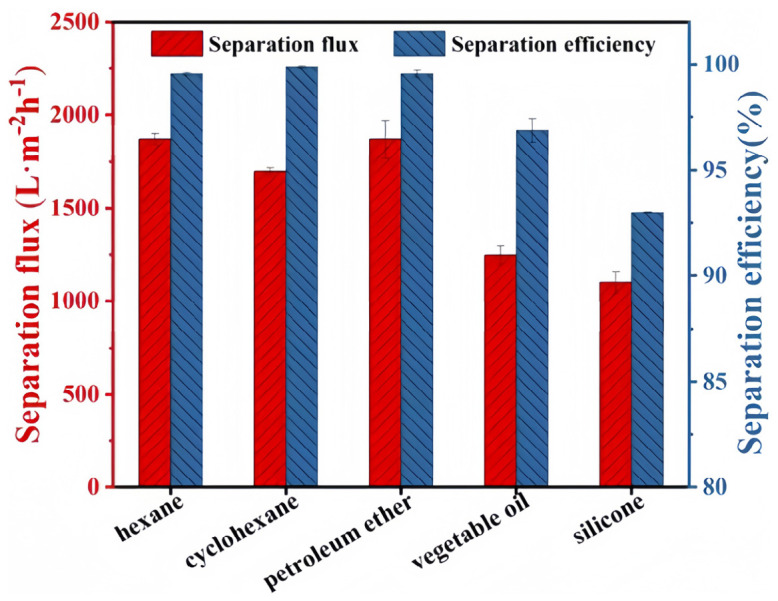
Permeation flux and separation efficiency of various oil-in-water (O/W) emulsions for optimal PPC20 membrane under gravity-driven conditions.

The permeation flux and oil rejection rate are critical parameters for evaluating the emulsion separation performance. As shown in Figure 8, the PPC20 membrane achieved outstanding separation for low-viscosity oils (hexane, cyclohexane, and petroleum ether), with oil rejection rates exceeding 99.5% and permeation fluxes above 1700 L m^−2^ h^−1^ under gravity-driven conditions. This demonstrates its high efficiency in separating low-viscosity O/W emulsions. In contrast, for emulsions made from vegetable and silicone oils, both the flux and separation efficiency showed a slight decline. This drop is likely due to the higher viscosity of these oils, which tend to produce more stable emulsions with finer droplet sizes. Consequently, the demulsification process required more time, resulting in a reduced permeation flux.

The optimal membrane for emulsion separation was defined based on three quantitative criteria: the shortest water spreading time (complete wetting within 5 s, Figure 3c), the highest underwater oil contact angle (158°, Figure 4), and the highest permeation flux (>2300 L m^−2^ h^−1^) while maintaining a separation efficiency of >99.5% (Figure 8). Based on these criteria, PPC20 was identified as the optimal formulation.

### 3.5. Dye Adsorption Performance

**Figure 9:**
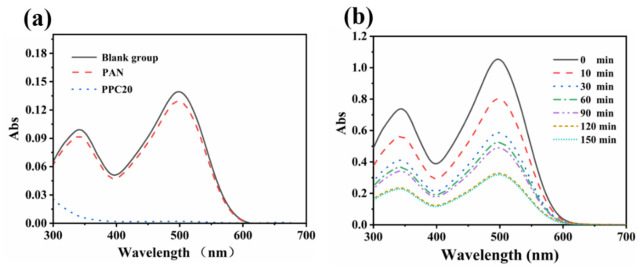
(**a**) UV–vis absorption spectra of Congo red (CR) solution before and after adsorption by the pristine PAN and the optimal PPC20 membranes. (**b**) Time-dependent UV–vis absorption spectra of the CR solution during adsorption on the PPC20 membrane.

Organic dyes are a class of persistent pollutants in industrial wastewater that are resistant to natural degradation, posing a significant challenge to conventional treatment processes [42]. Therefore, evaluating the dye adsorption capacity of membrane materials is crucial for their potential application in complex wastewater purification processes. The Congo red (CR) adsorption performance of the optimal PPC20 membrane was evaluated and compared with that of the pristine PAN membrane. As shown in Figure 9a, the UV–vis spectrum of the CR solution after filtration through the pristine PAN membrane was nearly identical to that of the original solution, with a negligible reduction in absorbance at the characteristic wavelength of 498 nm. In contrast, the UV–vis absorbance of the filtrate from the PPC20 membrane decreased to nearly zero, indicating effective CR adsorption during filtration.

To investigate the adsorption kinetics, 10 mg of the PPC20 membrane was immersed in 10 mL of a 50 mg L^−1^ CR solution and shaken at 35 °C and 150 rpm. The solution samples were taken at pre-determined time intervals and filtered through a 0.45 μm microporous membrane, and the residual dye concentration was analyzed using a UV–vis spectrophotometer. As shown in Figure 9b, the absorbance of the CR solution decreased gradually with increasing adsorption time. The adsorption capacity of the PPC20 membrane was high, and the absorbance of the 450–600 nm wavelength range was close to zero, indicating that the dye was almost completely removed from the solution. The good CR adsorption performance of PPC20 is mainly attributed to the rich active functional groups (amino -NH_2_ and hydroxyl -OH) on the PVA-CS layer, which can interact with anionic CR molecules via electrostatic attraction, hydrogen bonding, and van der Waals forces.

Adsorption experiments were performed to assess the Congo red (CR) removal performance of the as-prepared membranes. The corresponding kinetic fitting results are shown in Figure S5 and Tables S1 and S2. The pseudo-second-order kinetic model exhibited the best agreement with the experimental data, with correlation coefficients exceeding 0.99. These results suggest that CR adsorption onto PPC30 is predominantly controlled by chemisorption.

**Figure 10:**
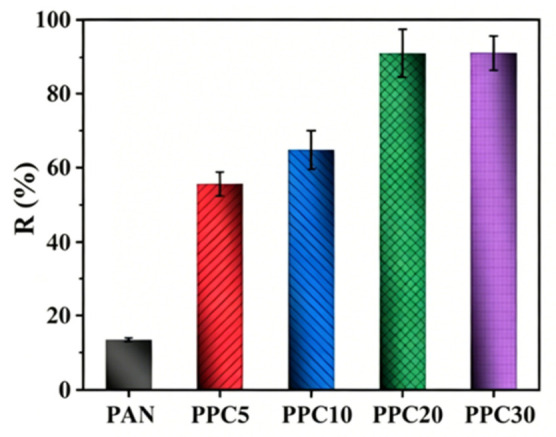
Congo red (CR) removal efficiencies of membranes with different CS contents.

Although PPC20 exhibited the best emulsion separation performance (Section 3.3), the adsorption mechanism was investigated using PPC30, which had the highest CS content. This choice was based on two considerations. First, PPC30 provides the maximum density of amino and hydroxyl functional groups. Second, the comparable adsorption capacities of PPC20 and PPC30 (Figure 10) suggest that the mechanism insights obtained from PPC30 can be reasonably extended to PPC20.

Under identical conditions, the adsorption performance of pure PAN, PPC5, PPC10, PPC20 and PPC30 membranes was compared. The pure PAN membrane exhibited an extremely low adsorption capacity of approximately 7 mg g^−1^ toward CR. The adsorption performance was remarkably improved with increasing chitosan (CS) content, and the PPC30 membrane achieved the maximum equilibrium adsorption capacity of 61.3 mg g^−1^. The similar adsorption capacities of PPC20 and PPC30 can be explained by the saturation of accessible surface active sites. Excess CS failed to provide additional active sites due to steric hindrance [43]. The CR removal efficiencies of membranes with different CS contents are displayed in Figure 10. The removal efficiency increased with CS content and then remained stable when the CS content exceeded 20%, which further verifies the saturation of adsorption sites [43,44].

### 3.6. Simultaneous Separation of Dye-Containing O/W Emulsions

The primary objective of this study is to develop multifunctional membranes capable of simultaneously removing oil droplets and dissolved dyes from complex wastewater. Accordingly, the performance of the PPC20 membrane for treating a hexane-in-water emulsion containing Congo red (CR) was evaluated using the PVA-CS layer as the separating surface.

**Figure 11:**
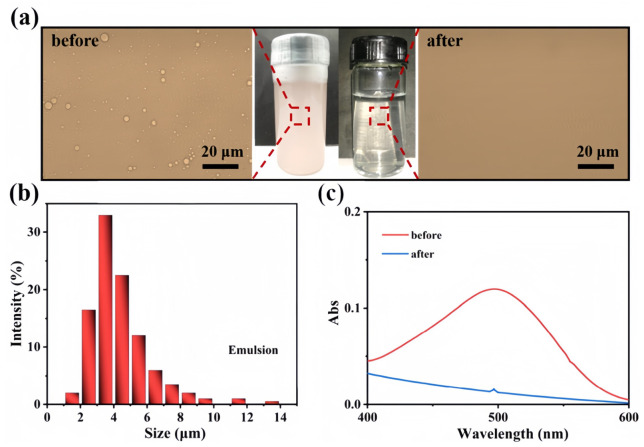
(**a**) Digital images and corresponding optical micrographs of the Congo red (CR)-containing oil-in-water (O/W) emulsion before and after gravity-driven filtration using the optimal PPC20 membrane. (**b**) Droplet size distributions and (**c**) UV–vis absorption spectra of the emulsion before and after separation.

As shown in Figure 11a, the initial pink and milky emulsion became clear and colorless after gravity-driven separation. Optical microscopy revealed numerous tiny oil droplets in the feed emulsion but none in the filtrate. Droplet size distribution analysis (Figure 11b) showed that the original emulsion had a narrow size range centered around 3.5 μm, whereas no droplets were detected in the filtrate, indicating excellent emulsion separation. In addition, the UV–vis spectrum of the filtrate (Figure 11c) displayed no characteristic absorption peak for CR, confirming that the dye was completely removed during the separation process. This effective simultaneous separation is the consequence of a synergistic mechanism in which the numerous active functional groups on the PVA-CS layer adsorb CR molecules while the excellent underwater oleophobicity hierarchical micro/nanostructure of the membrane physically inhibits oil droplets.

### 3.7. W/O Emulsion Separation Performance

**Figure 12:**
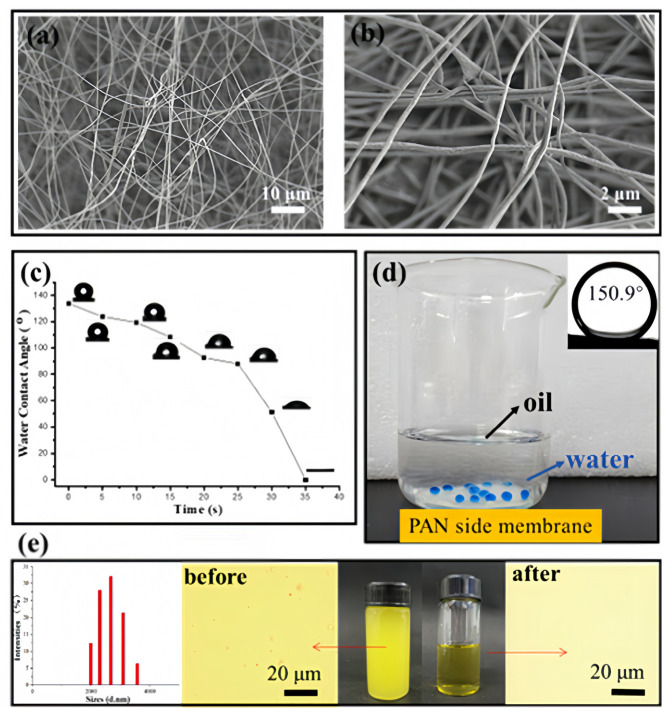
(**a**,**b**) FE-SEM images of the PAN layer in the optimal PPC20 membrane. (**c**) Time evolution of water contact angles (WCAs) on the PAN layer in air. (**d**) Under-oil water contact angle (UOWCA) of the PAN layer in hexane (inset shows the optical image of a water droplet). (**e**) Digital images, corresponding optical micrographs, and droplet size distributions of the water-in-oil (W/O) emulsion before and after gravity-driven separation.

The prepared PAN/PVA-CS bilayer membrane exhibited asymmetric wettability. Consequently, its performance in separating water-in-oil (W/O) emulsions was evaluated using a hydrophobic PAN layer as the separation surface. The surface morphology of the PAN side of the optimal PPC20 membrane was characterized using FE-SEM (Figure 12a,b). The PAN fibers exhibited a uniform, bead-free structure with distinct surface wrinkles and an average diameter of approximately 278 nm. This hierarchical fibrous architecture provides an appropriate pore size and considerable surface roughness, which is advantageous for achieving special wettability. The time-dependent water contact angle (WCA) of the PAN side in air is shown in Figure 12c. The initial WCA was 134°, confirming the inherent hydrophobicity of the PAN layer. However, owing to the high porosity and capillary wicking effect of the fibrous membrane, the WCA gradually decreased and reached 0° within 35 s. The under-oil water contact angle (UOWCA) of the PAN side in hexane is shown in the inset of Figure 12d, measuring approximately 151°, which demonstrates the excellent under-oil superhydrophobicity of the surface. This under-oil superhydrophobicity was uniform across the entire membrane and remained stable over an extended period, as confirmed by visual observations. This unique wettability renders the PAN side suitable for efficient W/O emulsion separation.

The W/O emulsion separation performance was evaluated using a water-in-vegetable oil emulsion as a model system. The emulsion was prepared by adding 1 mL of distilled water to 99 mL of vegetable oil, followed by sonication for 1 h to obtain a uniform milky-yellow emulsion. The membrane was pre-wetted with vegetable oil and assembled in a custom gravity-driven separation device with the PAN layer facing the feed solution. The as-prepared W/O emulsion was poured onto the PAN side, and separation proceeded solely under gravity. The results are presented in Figure 12e. The original milky-yellow emulsion became clear and transparent after separation. Under an optical microscope, the feed emulsion showed many tiny water droplets at the microscale, but no such droplets were observed in the filtrate. In addition, the filtrate contained no visible particulate matter, further supporting the high efficiency of the separation process. These findings indicate that the electrospun PAN/PVA-CS bilayer membrane has excellent bidirectional separation capability. It can effectively treat both dye-containing O/W and W/O emulsions by utilizing the different wettability of its two sides.

## 4. Conclusions

In summary, this study presents a facile and scalable sequential electrospinning strategy to fabricate PAN/PVA-CS bilayer membranes with asymmetric wettability, hierarchical micro/nanostructures, and integrated multifunctionality for complex wastewater treatment. By regulating the chitosan content in the PVA-CS layer, the membrane morphology, surface roughness, and functional group density were effectively optimized.

The optimized PPC20 membrane enabled gravity-driven, single-pass removal of both emulsified oils and dissolved dyes. Its PVA-CS surface exhibited excellent underwater oleophobicity with oil contact angles exceeding 158° and low oil adhesion, achieving O/W emulsion separation efficiencies above 99.5% with fluxes up to 2372 L m^−2^ h^−1^. Meanwhile, the membrane showed efficient adsorption toward Congo red, with a maximum equilibrium adsorption capacity of 61.3 mg g^−1^. Notably, the membrane also displayed bidirectional separability: when flipped, the hydrophobic PAN side, with an under-oil water contact angle of approximately 151°, efficiently separated W/O emulsions with efficiencies above 97.5%, eliminating the need for separate membranes or complex switching systems.

The robust bilayer architecture is attributed to intermolecular hydrogen bonding among chitosan, PVA, and PAN, which improves the spinnability and mechanical integrity of the chitosan-containing nanofibers while strengthening interlayer adhesion without chemical crosslinking or post-treatment. In addition, the PAN/PVA-CS membrane exhibited excellent stability under acidic, alkaline, and saline conditions, highlighting its potential applicability to harsh industrial wastewater environments.

Overall, the PAN/PVA-CS bilayer membrane combines simple fabrication, gravity-driven operation, high flux, oil/dye dual-function removal, bidirectional emulsion separation, and chemical robustness, offering a promising platform for scalable treatment of complex wastewater containing emulsified oils and soluble organic dyes.

## Data Availability

The original contributions presented in this study are included in the article. Further inquiries can be directed to the corresponding author.

## Supplementary Materials

The supporting information can be downloaded at https://www.mdpi.com/article/10.3390/membranes16070224/s1. Figure S1: SEM images of (a) PAN surface, (b) the cross section and (c) PVA-CS surface of PAN/PVA-CS membrane. Figure S2: (a) Digital images showing the spreading behavior of water and oil droplets on the PVA-CS surface of PPC20 in air. (b) Photographs of oil droplets on the PVA-CS surface of PPC20 under water, demonstrating its underwater superoleophobicity. (c) Photographs of the as-prepared large-area electrospun PAN/PVA-CS bilayer membrane (approximately 50 cm × 30 cm). Figure S3: Underwater dynamic oil adhesion behavior on the PVA-CS surface of the PPC20 membrane. Figure S4. (a) Standard calibration curve of Congo red (CR) concentration. (b) Equilibrium adsorption capacity and removal efficiency of CR on PPC30 as a function of initial concentration. Figure S5. (a) Adsorption kinetics of CR on PPC30 at different initial concentrations. (b, c) Pseudo-first-order and pseudo-second-order kinetic model fitting for the data in (a). (d) Adsorption kinetics of CR on membranes with varying CS contents (PAN, PPC5, PPC10, PPC20, and PPC30). (e, f) Pseudo-first-order and pseudo-second-order kinetic model fitting for the data in (d). Table S1. Kinetic parameters for Congo red (CR) adsorption onto PPC30 at different initial concentrations. Table S2. Kinetic parameters for Congo red (CR) adsorption on membranes with varying chitosan (CS) contents.
