# Supplementary material for: Electrospun PAN/PVA-CS Membranes with Asymmetric Wettability for Simultaneous Emulsion Separation and Dye Removal
Source: Membranes (Basel). 2026 Jun 29;16(7):224. doi: 10.3390/membranes16070224 (PMC13413893; doi:10.3390/membranes16070224)
Supplement: Supplementary file 1 [file membranes-16-00224-s001.zip › membranes-4362736-supplementary.pdf]

Supplementary Materials

Electrospun PAN/PVA-CS Membranes with Asymmetric Wettability for Simultaneous Emulsion Separation and Dye Removal

Tengfei Liao †, Zengpeng Zhang †, Qingxia Zhang and Hao Yang *

Key Laboratory for Green Chemical Process, Ministry of Education, School of Environmental Ecology and Biological Engineering, Wuhan Institute of Technology, Wuhan 430205, China

* Correspondence: hyang@wit.edu.cn

† These authors contributed equally to this work.

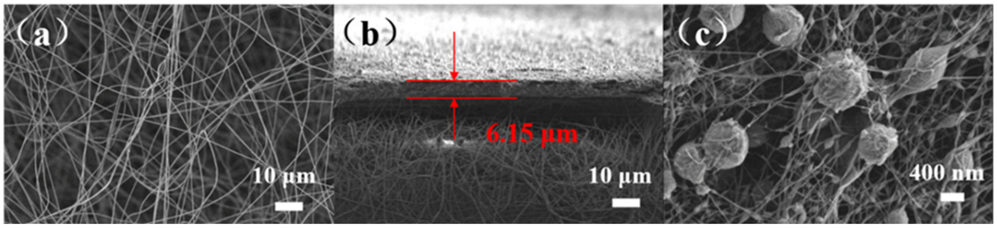

Figure S1. SEM images of (a) PAN surface, (b) the cross section and (c) PVA-CS surface of PAN/PVA-CS membrane.

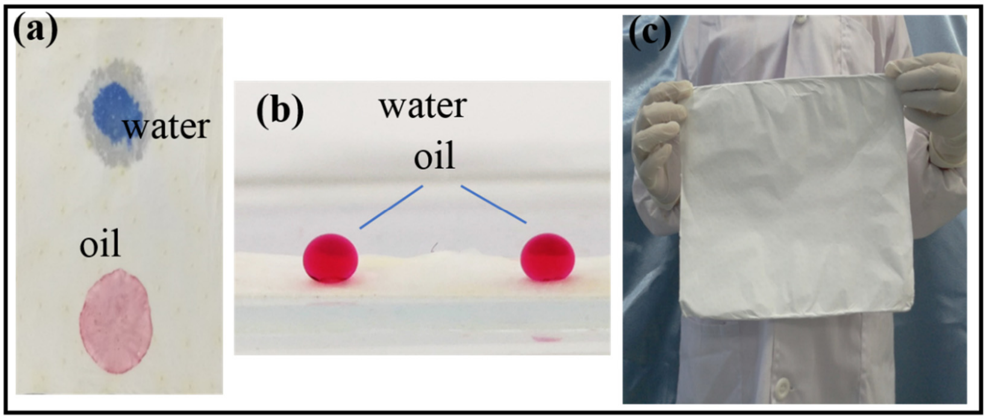

Figure S2. (a) Digital images showing the spreading behavior of water and oil droplets on the PVA-CS surface of PPC20 in air. (b) Photographs of oil droplets on the PVA-CS surface of PPC20 under water, demonstrating its underwater superoleophobicity. (c) Photographs of the as-prepared large-area electrospun PAN/PVA-CS bilayer membrane (approximately 50 cm × 30 cm).

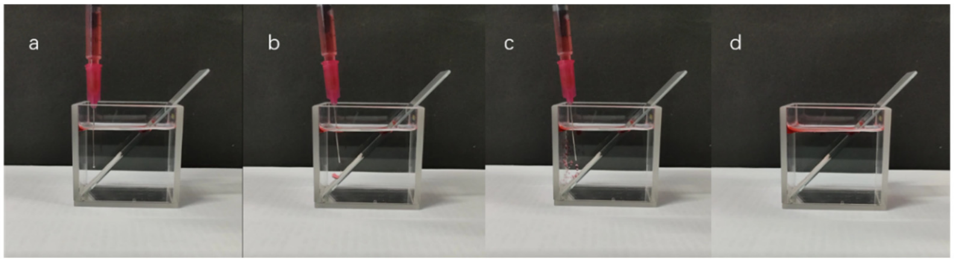

Figure S3. Underwater dynamic oil adhesion behavior on the PVA-CS surface of the PPC20 membrane.

Adsorption Kinetics Studies

The adsorption kinetics data were fitted using the pseudo-first-order (Equation S1) and pseudo-second-order (Equation S2) kinetic models:

$$\ln(Q_e - Q_t) = \ln Q_e - K_1 t \quad \text{(S1)}$$

$$\frac{t}{Q_t} = \frac{t}{Q_e} + \frac{1}{K_2 Qe^2} \quad \text{(S2)}$$

where Q_t (mg g^{-1}) is the adsorption capacity at time t (min), and k_1 (min^{-1}) and k_2 (g mg^{-1} min^{-1}) are the rate constants of the pseudo-first-order and pseudo-second-order models, respectively.

To elucidate the adsorption mechanism of Congo red (CR) on the membranes, systematic kinetic adsorption studies were conducted. The standard curve for CR quantification is provided in Figure S4a. As shown in Figure S5a, the equilibrium adsorption capacity increased with higher initial CR concentration until saturation, whereas the CR removal efficiency decreased with increasing concentration (Figure S4b).

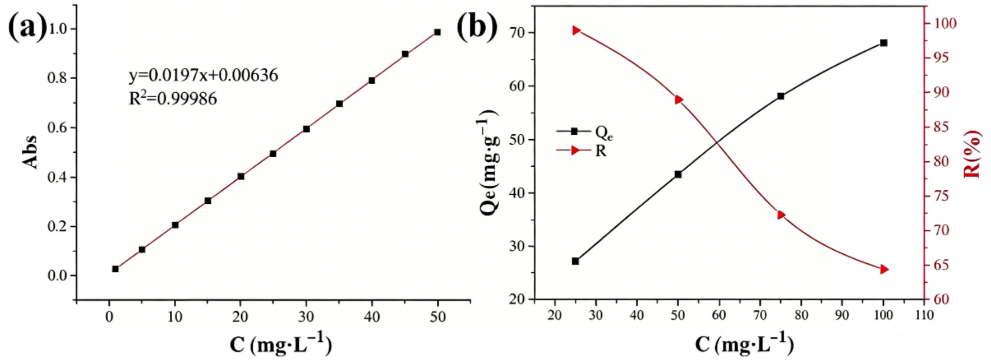

Figure S4. (a) Standard calibration curve of Congo red (CR) concentration. (b) Equilibrium adsorption capacity and removal efficiency of CR on PPC30 as a function of initial concentration.

The adsorption process could be divided into three stages. During the initial stage (0-30 min), the adsorption capacity increased rapidly, mainly owing to the high initial dye concentration and the abundance of available active sites on the membrane surface. In the second stage (30-150 min), the adsorption rate gradually slowed down as the CR

concentration in the solution decreased and most of the surface active sites became occupied. After 150 min, the adsorption capacity reached a plateau, indicating that adsorption-desorption equilibrium had been established. Therefore, 150 min was selected as the equilibrium time for the subsequent adsorption experiments.

The adsorption kinetics were evaluated by applying both pseudo-first-order and pseudo-second-order models. According to the results presented in Figures S5b and S5c, along with the data summarized in Table S1, the pseudo-second-order model provided a better fit to the experimental data, showing higher correlation coefficients ($R^2 > 0.99$) across all tested concentrations. This finding indicates that the CR adsorption of PPC30 is mainly controlled by chemisorption, which involves chemical interactions between the dye molecules and the functional groups present on the adsorbent surface.

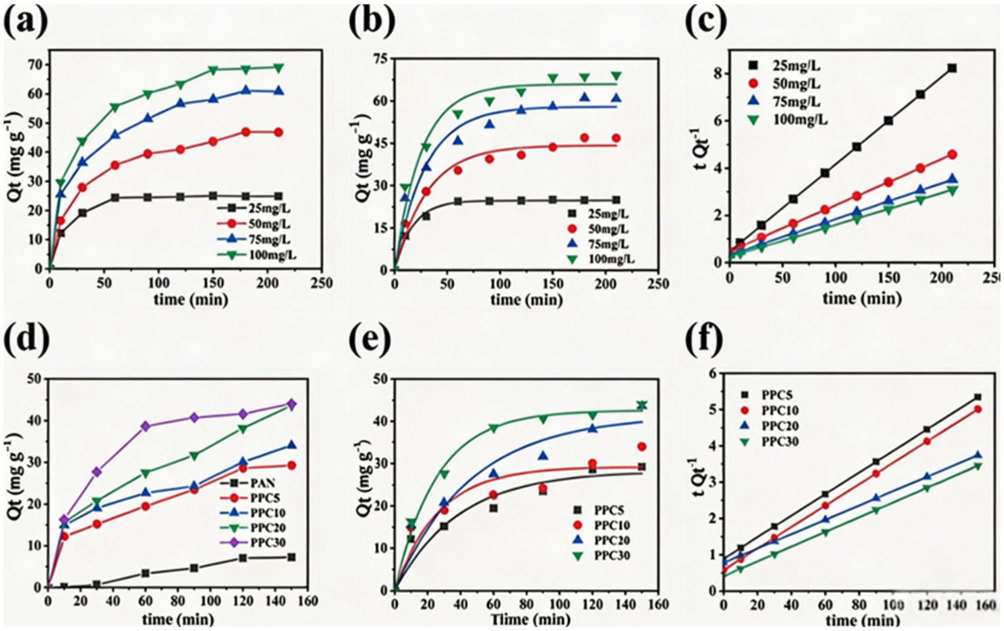

Figure S5. (a) Adsorption kinetics of CR on PPC30 at different initial concentrations. (b, c) Pseudo-first-order and pseudo-second-order kinetic model fitting for the data in (a). (d) Adsorption kinetics of CR on membranes with varying CS contents (PAN, PPC5, PPC10, PPC20, and PPC30). (e, f) Pseudo-first-order and pseudo-second-order kinetic model fitting for the data in (d).

Table S1. Kinetic parameters for Congo red (CR) adsorption onto PPC30 at different initial concentrations.

	Pseudo-first-order			Pseudo-second-order		
C ($mg \cdot L^{-1}$)	Q_e ($mg \cdot g^{-1}$)	K_1 (min^{-1})	R^2	Q_e ($mg \cdot g^{-1}$)	K_2 ($g \cdot mg^{-1} \cdot min^{-1}$)	R^2
25	24.76	0.0591	0.9927	27.00	0.0045	0.9986
50	44.27	0.0322	0.9711	51.26	0.0007	0.9963
75	57.98	0.0343	0.9541	66.03	0.0006	0.9967
100	65.90	0.0399	0.9655	74.40	0.0006	0.9982

The effect of CS content on CR adsorption was further studied by comparing the PAN, PPC5, PPC10, PPC20 and PPC30 membranes under identical conditions. As shown in Figure S5d, the pristine PAN membrane exhibited a very low adsorption capacity (~7 mg g^{-1}), demonstrating a negligible affinity for CR. In contrast, the adsorption capacity increased markedly with higher CS content, confirming the dominant role of CS in the adsorption process. The maximum equilibrium adsorption capacity of 61.3 mg g^{-1} was achieved by PPC30. The capacities of PPC20 and PPC30 were very similar, which is attributed to the saturation of accessible surface sites. Excess CS cannot provide additional active sites due to steric hindrance effects [1]. Kinetic fitting results for membranes with different CS contents (Figure S5e, f; Table S2) also confirmed that the adsorption process for all samples is best described by the pseudo-second-order model.

Table S2. Kinetic parameters for Congo red (CR) adsorption on membranes with varying chitosan (CS) contents.

	Pseudo-first-order			Pseudo-second-order		
Samples	Q_e (mg·g^{-1})	K_1 (min^{-1})	R^2	Q_e (mg·g^{-1})	K_2 (g·mg^{-1}·min^{-1})	R^2
PPC5	28.28	0.0281	0.9057	33.66	0.0008	0.9586
PPC10	29.27	0.0379	0.8604	33.89	0.0009	0.9423
PPC20	41.37	0.0218	0.9143	50.70	0.0004	0.9422
PPC30	41.90	0.0402	0.9873	49.33	0.0009	0.9978

References

1. Das, L.; Das, P.; Bhowal, A.; Bhattachariee, C. Synthesis of hybrid hydrogel nano-polymer composite using graphene oxide, chitosan and PVA and its application in waste water treatment. Environ. Technol. Innov. 2020, 18, 100664, doi:10.1016/j.eti.2020.100664.
